# Diseases Admitted under the Care of the Physicians of the Westminster Hospital, from the 20th of October to the 20th of November 1799

**Published:** 1799-12

**Authors:** 


					Difeafes admitted under the care of the Phyjlcians of the Wefiminjler llofti-'
tal, from the 20th of Ottober to the 20th of November 1799.
Fevers - - 13
Pleurify - 1
Scarlatina - - 2
Quinzey 2
? Amenorrhcea - -2
Anafarca 5
Afthenia - 5
Afthma 2
Catarrh - 1
Convulfions - - 1
Colica Piftonum - - 1
Colic - 1
Cough - - - 4
Cephala - 4
Diarrhoea - 3
Dyfpepfia - 5
DyfpncEa - 1
Dyfuria - 1
Enterodynia - - 2
Epilepfy - - 1
Epiftaxis - - - I
Gastrodynia 4
Hxmoptoe - 4
Ha:morrhois - 1
Hooping Cough - 1
Hypochondriafis - 1
Hyfteria - - 1
Impetigo - 6
Itch - 1
Lumbago - 3
Menorrhagia - 1
Phthifis - >- 2"
Paralyfis 3
Pleurodynia - ? 1
Rheumatifm - 6
Struma - -3
Tinea 2
Vomiting - 1
Worms - - 2

				

